# Blood Genomics Identifies Three Subtypes of Systemic Lupus Erythematosus: “IFN-High,” “NE-High,” and “Mixed”

**DOI:** 10.1155/2021/6660164

**Published:** 2021-07-01

**Authors:** Mintian Cui, Taotao Li, Xinwei Yan, Chao Wang, Qi Shen, Hongbiao Ren, Liangshuang Li, Ruijie Zhang

**Affiliations:** College of Bioinformatics Science and Technology, Harbin Medical University, Harbin 150086, China

## Abstract

**Purpose:**

Systemic lupus erythematosus (SLE) is a systemic and multifactorial autoimmune disease, and its diverse clinical manifestations affect molecular diagnosis and drug benefits. Our study was aimed at defining the SLE subtypes based on blood transcriptome data, analyzing functional patterns, and elucidating drug benefits.

**Methods:**

Three data sets were used in this paper that were collected from the Gene Expression Omnibus (GEO) database, which contained two published data sets of pediatric and adult SLE patients (GSE65391, GSE49454) and public longitudinal data (GSE72754) from a cohort of SLE patients treated with IFN-*α* Kinoid (IFN-K). Based on disease activity scores and gene expression data, we defined a global SLE signature and merged three clustering algorithms to develop a single-sample subtype classifier (SSC). Systematic analysis of coexpression networks based on modules revealed the molecular mechanism for each subtype.

**Results:**

We identified 92 genes as a signature of the SLE subtypes and three intrinsic subsets (“IFN-high,” “NE-high,” and “mixed”), which varied in disease severity. We speculated that IFN-high might be due to the overproduction of interferons (IFNs) caused by viral infection, leading to the formation of autoantibodies. NE-high might primarily result from bacterial and fungal infections that stimulated neutrophils (NE) to produce neutrophil extracellular traps (NETs) and induced individual autoimmune responses. The mixed type contained both of these molecular mechanisms and showed an intrinsic connection.

**Conclusions:**

Our research results indicated that identifying the molecular mechanism associated with different SLE subtypes would benefit the molecular diagnosis and stratified therapy. Moreover, repositioning of IFN-K based on subtypes also revealed an improved therapeutic effect, providing a new direction for disease treatment and drug development.

## 1. Introduction

Systemic lupus erythematosus (SLE) is a systemic multifactorial autoimmune disease, with breaches of tolerance in both T cells and B cells. Pathological T cell-B cell interactions and the production of autoantibodies are hallmark features of SLE [1]. The activation of B and T cell immunity (i.e., the adaptive immune system) requires the support of the innate immune system. A basic family required for recognition by the innate immune system is the Toll-like receptor (TLR) family. TLRs can quickly recognize a series of related molecular patterns found on bacteria, viruses, and fungi. The recognition of these molecular patterns on pathogens can trigger the production of proinflammatory cytokines of patients [2]. Proinflammatory cytokines not only participate in adaptive immunity and drive T cell activation but also can stimulate extramedullary hematopoiesis leading to expansion of innate immune cells. When autoantibodies are generated in an inflammatory milieu, they will be transformed into pathogenic isotypes. Excessive accumulation of autoantibodies can cause tissue damage in SLE [3].

SLE involves complex molecular processes, many of which are uncharacterized. Genetic interactions with infection, especially viral infection, might trigger the disease, leading to immune dysregulation [4]. Patients from non-European populations, such as Hispanics, African Americans, and Asians, are predisposed to develop the condition and progress more rapidly [5, 6]. Currently, the etiology and pathogenesis of SLE are unknown, and personalization of treatment remains challenging.

Recent identification of molecular disease subtypes based on clinical symptoms, disease severity, and pathogenesis has advanced our understanding of molecular processes in SLE [7–9]. Lanata et al. identified three lupus clinical subtypes defined by the ACR classification criteria that vary according to disease severity. They also found that patterns of differential methylation are associated with the subtypes and showed significant enrichment of genes associated with type I interferon signaling, antiviral responses, and inflammatory pathways. Banchereau et al. grouped patients by integrating gene expression information into functional modules. The functional modules showed IFN and neutrophil elastase (NE) characteristics. These works provide a framework for further study and have potential implications for the clinical therapy of SLE. However, even though different studies have shown similarities in subtypes, they have not reached a consistent conclusion, which emphasizes that we should conduct further research on its characteristics and mechanisms.

Patients show unique transcriptional characteristics linked to IFN and NE [10]. Cells use pathogen recognition receptors (PRR) such as TLRs to recognize pathogen-associated molecular patterns (PAMPs), and their activation initiates the release of IFN. This in turn leads to the release of proinflammatory cytokines, which can lead to autoimmunity [11]. A specific form of neutrophil cell death is called NETosis, which causes the release of neutrophil extracellular traps (NETs). NETs with potential immunogenicity cause autoimmunity via multiple pathways [12].

SLE is primarily treated with hydroxychloroquine (HC), corticosteroids (CSs), and immunosuppressive agents such as mycophenolate mofetil (MMF) and cyclophosphamide [13]. CSs are one of the most effective treatments for immediate relief of inflammation. However, long-term use of CSs may cause serious side effects. There are now data supporting a central role for the plasmacytoid dendritic cell-derived type I IFN pathway in SLE, and a number of antitype I IFN therapeutics have undergone evaluation in clinical trials. However, as with other therapeutics, there are some patients who do not apparently respond [14]. These reports on therapy of SLE suggested that response to drugs varies according to subtype [15], and stratified treatments guided accordingly may provide better outcomes.

Although researchers have developed these new therapies for SLE in the past ten years, they still lag behind other autoimmune diseases such as rheumatoid arthritis and Crohn's disease [16]. Data mining based on big data will transform our understanding of SLE and help develop new therapies. There have been many studies [7–9]. They have used different bioinformatics methods to mine the potential information in big data sets. Furthermore, if these large data sets are integrated for restudy, data can be fully utilized, thereby improving clinical practice [17]. However, this method also has limitations. First, the limitations of the data itself make it difficult for us to obtain the ideal data. Second, the generation of data-driven hypotheses needs to be verified again. Third, it is difficult to find a new perspective to explain the data, and there are limitations in timeliness. Therefore, it will be a challenge to select the appropriate data and combine the methods for in-depth information mining.

In this study, we aimed to identify an SLE global signature based on transcriptome analysis of two large published patient cohorts and develop a single-sample typing method. We aimed to reveal the molecular mechanism of each subtype through building a coexpression network of biological functions and provide a rationale for the stratified therapy of patients and drug development.

## 2. Method

### 2.1. Patient Populations

We used two unique data sets of pediatric and adult SLE patients traced over time. Both sets of samples were based on platform GPL10558 (Illumina HumanHT-12 V4.0 expression beadchip, San Diego, CA, USA), and clinical variables and genome-wide gene expression levels were measured at different time points for every patient. Gene expression data were downloaded from NCBI GEO (GSE65391, GSE49454). The GSE65391 data set is follow-up data of 158 pediatric patients, including 996 samples of which 72 are healthy samples and 924 are SLE samples. The data set includes the patient's age, gender, race, current treatment, time of illness, number of follow-ups, follow-up interval, SLEDAI score, and its 24 components at all time points. The GSE49454 data set is follow-up data of 62 adult patients, including 177 samples of which 20 are healthy samples and 157 are SLE samples. Clinical assessment, current treatment, laboratory results, and SLEDAI score were collected at each visit. In order to study the impact of different treatments on patients, we categorized drugs as immunosuppressive agents, CSs, and HC. For validation, patients were divided into discovery and test sets, using a ratio of 2 : 1. To obtain reliable genes related to disease activity and better evaluate drug benefits, patients with missing clinical data or who had drugs were changed during the test or SLEDAI [18] scores that remained stable over time were not included in the final analysis. In fact, only four patients (SLE-105, SLE-136, SLE-326, and SLE-59) whose SLEDAI scores remained stable over time were excluded, which would have had minimal effects on the results. A total of 405 samples (112 patients) were selected for further analysis, each one with a variable number of visits, continuous and categorical clinical variables, and gene expression data. The two cohorts did not have significant differences in distributions of race, gender, or treatment. Characteristics of the cohorts are summarized in Supplementary Table 1.

### 2.2. Processing of the Data

Quantile standardization is used to integrate transcripts shared by two sets of data. Transcripts with standard deviation below 0.1 across samples were removed. The reserved transcripts were annotated as gene symbols. Duplicated genes were merged assigning them their mean expression value. 405 samples and 203 samples were selected as the training set and the test set, respectively.

### 2.3. Screening of Blood SLE Signature

For each patient, we obtained gene expression data across a number of visits, as well as related SLEDAI scores and clinical variables measured in each visit. The blood SLE signature in the discovery set was determined as follows:

1. *Screening of the SLE differential genes based on an ANOVA model*: using an ANOVA model, the difference distribution was calculated to identify differentially expressed genes (DEGs) between SLE patients and healthy controls.
2. *Further screening of the genes related to disease activity scores*: based on the differential genes, we calculated the correlation between disease activity scores and longitudinal gene expression information. Taking into account the imbalance of the data (repeated measurements and missing records), we evaluated the weight of each patient. The DEGs were ranked by absolute correlation values, and the sum of all patients was calculated to obtain a unique score value for each DEG. We get the DEGs related to disease activity as the SLE global signature.
3. *Validating the signature of SLE*: first, we verified the correlation values between disease activity score and gene expression. By changing the rows and columns of gene expression matrices 1,000 times randomly, the correlation values were recalculated to conduct a permutation analysis. In addition, the SLE signature was also verified by relative literatures.

### 2.4. Discovery of the Single-Sample Classifier

Based on the SLE signature, we developed a single-sample subtype classifier (SSC) to predict consensus subtypes. The unsupervised clustering approaches (NbClust [19], PAM [20], and Vegan [21]) were used to determine the optimal number of subtypes and assign a category label for each sample. After calculating the gene expression pattern of the consensus class, an iterative algorithm was preformed to correct subtypes until convergence. Compared with the samples before correction, the SSC and subtype expression patterns were tested. We then calculated the SLEDAI level of each subtype before and after correction and compared the difference between subtypes.

### 2.5. Functional Analysis of the Subtype

To explain the subtypes functionally, we performed functional enrichment analysis of Gene Ontology terms in the expression of the signature in different subtypes. We used the gene expression deviation (GED) method [22] to measure the activity of terms. By identifying the differential genes between subtypes and healthy controls, we analyzed the pathway activity and functional patterns of different subtypes. To reveal the mechanism of subtypes, we chose to further analyze the regulation mechanism of subtypes in the mixed type.

### 2.6. Analysis of Treatment Effects

To assess whether the subtypes benefited from different treatments, we analyzed the changes in SLEDAI before and after each patient took different drugs in different subtypes. Because patients are almost always treated by combination medication, we only consider the changes in SLEDAI between patients who take a certain drug and those who do not take a certain drug and compare the changes in three subtypes.

### 2.7. Repositioning of Targeted Drug within Subtypes

Because subtypes show different core-driven patterns, we tried to explore whether new targeted core drugs showed different therapeutic effects in different subtypes. We used the public longitudinal data from Ducreux et al. [23]. The study included twenty-eight patients with SLE, according to the ACR criteria for SLE. Patients were randomized to receive three or four injections of placebo (*n* = 7) or 30 *μ*g (*n* = 3), 60 *μ*g (*n* = 6), 120 *μ*g (*n* = 6), or 240 *μ*g (*n* = 6) of IFN-*α* Kinoid (IFN-K). Gene expression data are taken from the GSE72754 download. We use SSC to classify patients and compare the benefits of different subtypes to IFN-K.

## 3. Results

### 3.1. The Global SLE Signature

**Figure 1:**
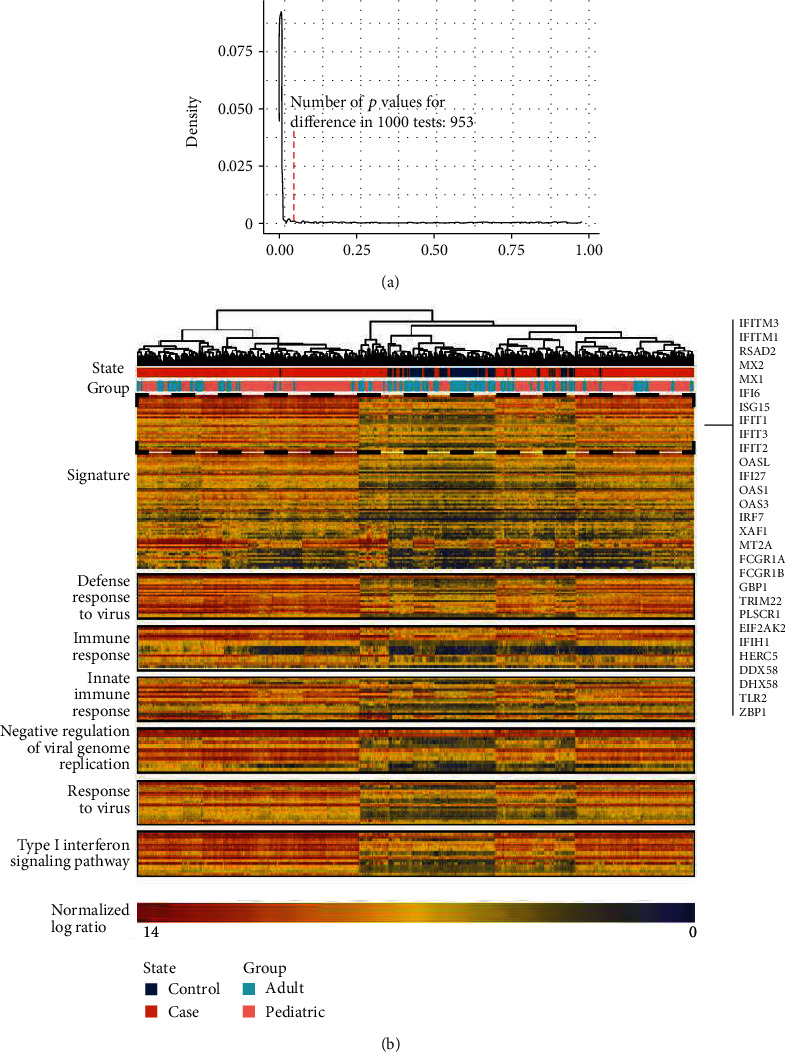
Analysis of signature in discovery set. (a) Correlation score permutation analysis. Null distribution generated by randomly permuting the expression profile 1000 times and identifying the number of *p* values for differences that were also significant (*p* < 0.05). The red line indicates the number of *p* values for significant differences in 1000 tests. (b) Heat map of signature in discovery set. 405 samples composing the SLE cohort were clustered using 92 genes. The column annotations across the top provide the distribution of the two queues (Group, State), and rows are fixed by biologically relevant gene sets. The heat map shows that samples from one data site are not clustered together, indicating limited batch effects.

We used disease activity scores and gene expression data to identify the global SLE signature. First, we defined DEGs in the discovery set by comparing all patient samples with healthy controls. Using an ANOVA model, we identified 113 DEGs (FDR < 0.05). Next, we analyzed the patient cohort longitudinally and obtained the correlation score between the gene and SLEDAI. To verify the correlation score, a permutation analysis was conducted by changing the rows and columns and testing the difference with the original score.  Figure 1(a) displays the density of different *p* values in 1000 tests. This analysis revealed that the score between genes and disease activity is reliable (*p* < 0.05). We randomly selected 113 nondifferent genes to calculate the score and compare it with the score of DEGs for comparison. In 1000 comparisons, the average score of DEGs was always higher than that of randomly selected genes and showed statistical significance (*p* = 0.008), indicating that genes related to disease activity levels are consistent with differential genes as a whole. Finally, we further screened genes by correlation score and difference distribution (FDR < 0.05, score > 0.6), and 92 genes were used as an overall SLE signature. These signature genes are not only differential genes between health and patients but also significantly related to the severity of patients.

Of these 92 genes, 72 (78%) have been verified as associated with SLE, mostly confirmed by multiple studies and experimental approaches. For example, real-time expression levels of IFIT2 are associated with SLE disease activity [24]. Genes that have not been verified in the literature are also related to autoimmune diseases. For example, TDRD7 is proven to be a key gene for Sjogren syndrome [25].

We perform hierarchical clustering and functional enrichment of the discovery set samples according to the expression values of signature genes (Figure 1(b)). Patients and healthy controls showed significant differences. Genes were significantly enriched in viral infections, excessive IFN activation, bacterial and fungal infections, and immune disorders (FDR < 0.01), implicating infection and IFN as pivotal factors in the induction of autoimmunity. We also found 30 (33%) prevalent IFN signature genes, indicating that IFN plays an important role in SLE.

### 3.2. Single-Sample Classifier to Predict SLE Subtypes

**Figure 2:**
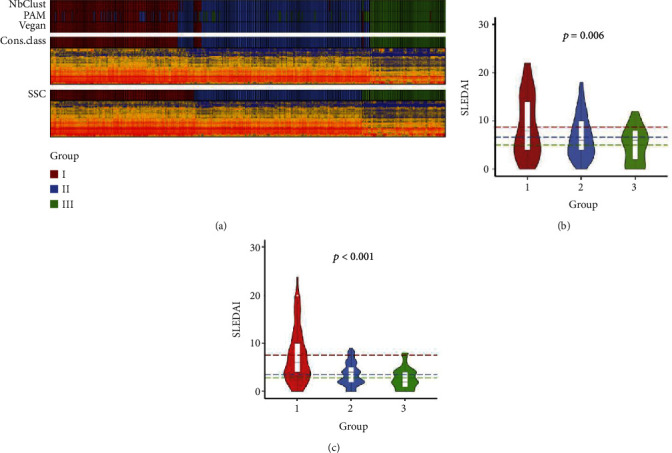
Molecular subtypes in SLE samples in the discovery set. (a) The model was trained to predict three classes. NbClust, PAM (partitioning around medoids), and Vegan reveal that the optimal number of subtypes is three. Calling these three algorithms to classify patient samples, the results showed strong consistency. After further correction based on the consensus category, we got a more accurate result. (b) SLEDAI in the subtypes after correction. There are differences in the mean values of the three subtypes of SLEDAI, and the differences of overall and between subtypes are significant, reflecting the accurate allocation of samples after correction. (c) SLEDAI in the subtypes in the validation set.

A single sample model was trained to predict subtypes using all SLE samples in the discovery set. The consensus category is determined by three algorithms based on different measures (Figure S1), and the model is further corrected by determining subtype characteristics. We calculated the expression value of the subtype signature genes and the distance from the samples to the expression value and reclassified the subtypes according to the distance. After several iterations of correction, it was found that the model converged when only a few samples were changed. Compared with the consensus classes, the corrected result was more discriminatory in assigning individual patients to a definitive subtype (Figure 2(a)), and the patient's heat map showed clearer results. The single-sample subtype classifier (SSC) finally selected 92 signature genes to produce the feature values for the prediction of three subtypes (Supplementary Table 2). The distance from the sample to the subtype feature value indicated the likelihood of the sample belonging to each of the three subtypes.

To further explore the relationship between subtypes and severity, a comprehensive comparison of SLEDAI was performed on the subtypes. To increase the accuracy of the results, we performed outlier detection on the patients' SLEDAI and intercepted the data according to the density distribution. Before the correction, we observed that the SLEDAI was different among the three clusters (Kruskal–Wallis *p* = 0.006, Figure S2c), with cluster III being the least severe and cluster I the most severe. However, there was no significant difference between subtypes II and III (*p* = 0.061). After correction, the difference between the severity of subtypes was more pronounced (Kruskal–Wallis *p* = 0.006, *p*_II vs.III_ = 0.022; Figure 2(b)), further confirming the SSC accuracy. The clustering of patients and healthy controls revealed that before and after correction, subtype III tended to cluster with healthy patients, indicating that subtype III likely represented mild patients (Figure S2a, b).

SSC was validated using a validation set of 203 SLE patients. We used the subtype characteristics to classify the sample and recalculate the accuracy for the subtype category. The results show that the characteristics of our subtypes are accurate (FDR < 0.05). To validate the three subtypes, we compared the SLEDAI of the samples in the validation set and again found that the three subtypes vary according to disease severity (Kruskal–Wallis *p* < 0.001; Figure 2(c)). Thus far, we identified three subtypes of SLE related to severity and their characteristics, which showed strong robustness.

### 3.3. Subtypes Express Different Functional Modes

The 36 terms enriched by SLE signature genes could be summarized into four main functional modules: virus infection, bacterial infection, fungal infection, and immune disorder. At the same time, due to the important role of interferon in SLE, we listed IFN separately as a functional module and analyzed it simultaneously [26]. We compared different subtypes within the same module and found that in all modules, subtype I was the most severe, consistent with our previous conclusions. Interestingly, subtype II was more dysregulated in the virus-related modules than type III, and subtype III was more dysregulated in the bacteria-related modules (Figure S3). Combined with our previous conclusion that subtype II was more serious than subtype III, we speculated that viral infections were more serious in SLE patients.

**Figure 3:**
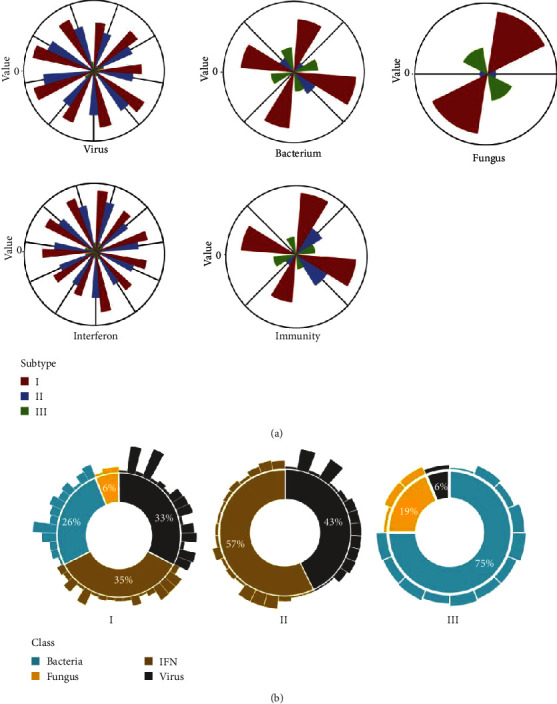
(a) Analysis of subtypes in functional modules. The function of disorders was divided into five modules: viral infection, bacterial infection, fungal infection, IFN disorder, and immune disorder. Each group of entries in the module represents BPs (biological processes), the height of the bars represents the degree of dysfunction, and the higher it is, the more serious the dysfunction. (b) Analysis of functional modules in subtypes. The middle circle represents the proportion of dysregulated functions in subtype, outer bars represent dysregulated functions, and the height of the bars represents the degree of dysregulation.

Next, we compared the different functional modes of the subtypes (Figure 3). To further verify our hypothesis, we redetermined the DEGs between subtypes and healthy controls and performed a functional analysis of each subtype. We found that subtype I was dysregulated in IFN and viral, bacterial, and fungal infections. Considering our previous conclusion that subtype I had the most serious dysregulation, we thought that subtype I was a mixed type of patient, affected by viral, bacterial, and fungal infections. We named this type “mixed.” Subtype II was only enriched in the viral infection and IFN dysregulation modules and showed a virus infection-related pattern with IFN as the core, which we named “IFN-high.” Subtype III was enriched in bacterial and fungal infection modules. It showed a bacterial and fungal infection-related pattern with NE as the core, which we named “NE-high.” In addition, it was observed that NE in NE-high was significantly higher (Wilcox test, *p* < 0.001) in both the child and adult groups, which supported our conclusion.

In functional modules gathered by the SLE signature, we found that the virus infection occupied a more important position, and in the separate analysis of subtypes, we again found that virus infection was more important. IFN-high was more severe compared to NE-high, which further supported the hypothesis that viral infections were more serious. In conclusion, we proposed that SLE subtypes were divided into three types: mixed, IFN-high, and NE-high, which were expressed in two patterns—one is a virus infection-related pattern with IFN as the core and the other is a bacterial and fungal infection-related pattern with NE as the core. Bacterial and fungal infections exhibited related patterns, and viral infections played a more important role in SLE [27].

### 3.4. Mechanisms of Immune Dysfunction and Their Connection

**Figure 4:**
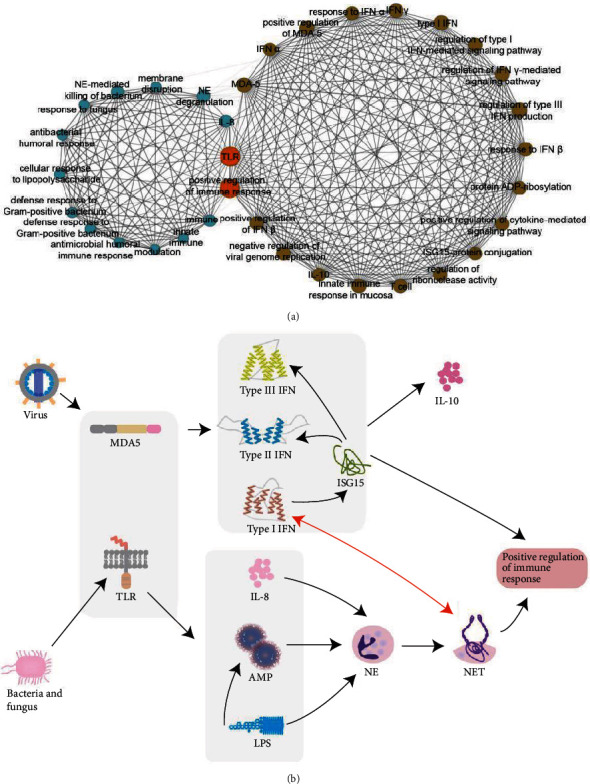
Mode mechanism of subtype. (a) Coexpression network of BPs in the mixed type. Each point in the network represents a kind of BP, the edges represent the correlation between functions, and the size of the edge represents the strength of the correlation. Points in the two communities are marked with different colors, and important bridge nodes are marked with orange. (b) Mechanism of immune dysfunction in mixed type. Viral infections and bacterial and fungal infections show different pathogenic pathways. Viral infections cause immune disorders by activating the overexpression of IFN; bacterial and fungal infections induce autoimmune reactions by inducing NE to produce NETs.

To further reveal the mechanisms of immune dysfunction, we constructed a BP-based coexpression network for the mixed type. The network was constructed based on the similarity of the functional terms, which was calculated based on the activity of the functional items in different patients (see the supplementary file). We also plotted a correlation coefficient graph (Figure S4). The coexpression network revealed two modules whose internal nodes were tightly connected to each other (Figure 4(a)), revealing that there are two different pathogenic mechanisms associated with the mixed type. After consulting the related literature, we determined that the items inside the modules showed functional connections. The two modules in the network were composed of IFN-related terms and NE-related terms, respectively, which represented two mechanisms in the mixed type, and were consistent with our expected results. Combined with the related literature and our research on subtype-specific functional modules, we have further connected these two mechanisms.

Through analysis of the community and consideration of the comprehensive background, we inferred two types of pathogenesis of SLE in the mixed type (Figure 4(b)). First, pathogen-associated molecular patterns (PAMPs) of the virus are recognized by the Toll-like receptors (TLRs) on the cell surface or in the endosome and the retinoic acid-inducible gene 1- (RIG-I-) like receptors (RLRs) in the cytoplasm, stimulating IFN production. Excessive accumulation of IFN leads to the transformation of almost all components of the immune system to pathological functions of tissue damage and disease development [28–30]. ISG15 is induced by type I interferon and acts as a bridge between it and type II and III interferon [31]. Both type I and type II interferons stimulate myeloid DCs (mDCs) to activate T and B cells, producing a variety of proinflammatory cytokines and autoantibodies. The increased production of IFN-*α* and IFN-*γ* by monocytes may trigger an inflammatory response, as well as the increased production of IL-10, leading to the secretion of autoantibodies by B cells in human SLE [32, 33]. Although type III interferon is functionally an interferon, its structure is similar to members of the IL-10 family and plays a protective role in viral infections [34].

In the second type of pathogenesis, bacteria and fungi are recognized by TLRs and promote the expression of the proinflammatory cytokine IL-8 [35]. The antimicrobial peptide (AMP) interacts with the lipopolysaccharide (LPS) to destroy the membrane structure and acts as an immune effect factor to initiate and regulate the autonomous immune system [36]. IL-8, AMP, and LPS, along with Gram-positive and Gram-negative bacteria and fungi, stimulate neutrophils to form extracellular structures called NETs to kill bacteria and fungi [37, 38]. NETs expose autoantigens, such as nucleic acids and proteins, in an inflammatory milieu that can stimulate an autoimmune response in a susceptible individual [39]. In addition, SLE NETs can activate pDC to produce high levels of IFN-*α* [40]. Similarly, IFNs act as priming factors on mature neutrophils, allowing the formation of extracellular traps upon subsequent stimulation with other factors [41]. We have verified two SLE model mechanisms within the IFN-high and NE-high groups.

### 3.5. Research on Age, Gender, and Race within Subtypes

To study the differences between child and adult patients, we grouped the samples according to age. We found that child patients were more inclined to be IFN-high and adult patients were more inclined to be mixed (Fisher's exact test, *p* = 0.028). Compared to adults, child exposure to viruses was significantly higher than adult exposure. This might be because the peripheral blood mononuclear cells (PBMC) from pediatric SLE patients are more sensitive to stimulation by transfection with viral RNA from the influenza virus [42]. This indicates that viral infection might play a more important role in the pathogenesis of SLE in children.

To study differences associated with gender, we grouped patients according to gender. We found that female patients were more inclined to be IFN-high and male patients were more inclined to be mixed (Fisher's exact test, *p* = 0.034). There was almost no deviation between the viral and bacterial types among men, but women were more likely to be affected by viruses. This might be due to the influence of estrogen on SLE disease activity. For a long time, it was believed that estrogen played a vital role in the occurrence and development of SLE [43], and women of childbearing age were more likely to develop SLE. Relevant studies have proven that overactivation of estrogen receptor-*α* (ER*α*) exacerbates lupus disease [44]. Further studies have determined that under the stimulation of TLR7 or TLR9, estrogen/ER*α* can promote pDC to secrete IFN-*α* [45]. This could explain why female patients are more inclined to be IFN-high and show more severe responses. Some studies also have reported that estrogen reduces the concentration of IL-8 in females [37], which might reduce the risk of the NE model of illness.

We divided the patients into black and white groups to assess racial differences. We found that white patients were more inclined to be mixed, and black patients were more inclined to be IFN-high (Fisher's exact test, *p* = 0.021). White patients showed almost no deviations between viruses and bacteria, while black patients were more likely to be affected by viruses and their disease was more severe. The influence of race on the clinical severity of SLE has been clearly described, with black patients usually having more severe disease manifestations than white patients [46].

### 3.6. Drug Treatment within Subtypes

Our study found that HC had the greatest effect on decreasing SLEDAI in the IFN-high subtype, followed by CSs and immunosuppressive agents. In the NE-high subtype, CSs had the greatest effect, followed by HC and immunosuppressive agents. In the mixed type, CSs had the greatest effect, followed by HC and immunosuppressive agents. Immunosuppressant agents exhibited a specific effect, which was consistent with previous studies [47]. However, it appeared that the effects of CSs were more distinct for some time periods. We found that molecular subtyping might have an impact on the benefit experienced from drug treatment. For example, HC has shown better effects in IFN-related subtypes, which has a specific promotion effect on the benefits of drug treatment, and its mechanism is worth further research. The latest research shows that CSs and HC might treat SLE through blockade of IFN [7, 48], which is consistent with our research. Subtype analysis revealed the benefits of different drugs and should be integrated into clinical studies as soon as possible.

We found that CSs continued to be the first line of treatment option for SLE. Due to their extensive anti-inflammatory and immunosuppressive effects, CSs play an irreplaceable role in SLE treatment. However, the required dose is high enough to have adverse effects, indicating that it is necessary to develop new drugs for the treatment of SLE.

### 3.7. Repositioning of IFN-K within Subtypes

To identify the therapeutic response of IFN-K to the three subtypes, we performed subtype relocation of IFN-K. IFN-K is a therapeutic vaccine that induces a polyclonal antibody (Ab) response that neutralizes all 13 human IFN-*α* subtypes. In patients who are IFN-signature positive, IFN-K significantly decreases the IFN gene signature. However, IFN-K failed to reach the primary clinical endpoint and did not show beneficial effect for some patients [23]. To relocate IFN-K within subtypes, we downloaded the data of GSE72754, which included the blood transcriptome data of 28 SLE patients. Extended follow-up data were collected in six of the 21 IFN-K-treated patients. We used 36 samples of these six patients for longitudinal analysis. After the cross-platform conversion of the sample expression data, SSC was used to classify the samples, and the degree of change in SLEDAI per unit time was calculated for the different subtypes. Among the 36 samples of 6 patients, 15 were of mixed type, 8 were of IFN-high type, and 13 were of NE-high type. The results showed that IFN-high received the greatest therapeutic effect (-0.333/month), followed by mixed (-0.120/month), while NE-high showed almost no therapeutic effect (-0.038/month). This was consistent with our expected conclusions and indicated that the repositioning of IFN-K within subtypes might have clinical benefits for patients.

## 4. Discussion

SLE is a highly heterogeneous autoimmune disease characterized by diverse clinical manifestations and varying degrees of severity. In this study, we developed an SSC based on a large integrated patient cohort and identified three subtypes of SLE that vary according to the severity of the disease. The molecular features of the subtypes reflected different functional mechanisms affecting patients. As the most serious subtype, the “mixed” was associated with two functional mechanisms which showed an inherent connection. IFN-high was a subtype with IFN as the core, characterized by a positive immune response caused by overexpression of IFN. The mildest subtype, NE-high was characterized by the generation of NETs induced by NE, leading to autoimmune responses due to autoantigen exposure. Another important finding of our analysis was that subtypes benefited from different drugs, which represents a major advancement in understanding the underlying molecular mechanisms and their potential clinical ramifications.

The latest molecular characterization of SLE, including the subtype description based on gene expression, provides a framework for further research of this common autoimmune disease. It also provides potential biological insights for the different clinical phenotypes [49, 50]. For example, in the original study of the pediatric patient data set, the researchers also identified IFN as an important marker of SLE and divided patients into seven subgroups. However, in that study, more attention was given to the identification of subgroup markers. We speculated on the different pathogenesis of each subtype and attempted to explain the subtype functionally. In the original study of the adult patient data set, the researchers reported complex IFN markers for SLE, which were not limited to the previous IFN-*α* markers but also involved IFN-*β* and IFN-*γ*, which was consistent with our findings. However, their study was more focused on patients with IFN abnormalities. In our study, we also paid attention to patients who did not show abnormal IFN and attributed them to “NE-high”.

As a key factor related to autoimmune responses (especially SLE), infection has been implicated numerous times as the primary trigger of these two mechanisms [51]. Viruses stimulate T cells to produce more IFN by binding to receptors. IFNs are key proinflammatory cytokines thought to be involved in the pathogenesis of SLE. Bacteria and fungi bind to TLRs on T and B cells and activate immune cells to produce the proinflammatory cytokine IL-8, which leads to autoimmunity by inducing NETs. Moreover, there is a close connection between IFN and NETs. Compared with bacterial and fungal infections, viral infections with IFN at the core exhibit a more important role in the occurrence and development of SLE.

In this study, we required that the data set used should have a larger sample size and detailed clinical follow-up data (including gender, age, race, SLEDAI, and blood transcriptional profiles). Finally, we selected two large patient cohorts based on the same platform, one of which was an adult patient data set and the other a child patient data set. We comprehensively analyzed the effects of age, gender, and race on patients and found that there are significant differences in the distribution of subtypes among people of different gender, age, and race. When considering age, children showed increased susceptibility to viral infections and had more severe disease than adult patients. Compared to males, female SLE patients are more inclined to belong to the IFN-high subtype of SLE. Interestingly, studies have found that under the stimulation of TLR7 or TLR9, estrogen/ER*α* can promote pDC to secrete IFN-*α*, which was associated with increased severity. As for race, black patients showed higher levels of IFN.

An important finding of our research was that distinct subtypes benefited from different drugs. CSs had the greatest effect on decreasing SLEDAI in the NE-high and mixed subtypes. IFN-high patients benefited from all drugs irrespective of treatment strategies, while HC showed the best effect. Immunosuppressant agents exhibited specific effects, which is consistent with previous studies. However, it appeared that the effect of CSs was more pronounced for some time periods. Thus, for patients' medication, physicians should include subtyping within the treatment selection as soon as possible.

Presently, novel targeted drugs have attracted widespread attention, but because some patients do not benefit from new therapies, many drugs' clinical development has been terminated [52]. Although IFN-K is a therapeutic vaccine that modulates the type I IFN pathway and has shown therapeutic effects in SLE patients, it has not reached the main clinical endpoint. In this study, we tried to reposition IFN-K within subtypes and revealed that the drug has the most therapeutic effect in IFN-high patients but almost no therapeutic effect in NE-high patients. This provides an important development for drug therapy: new targeted drugs should be rationally repositioned into stratified treatments. This may rejuvenate the use of existing targeted drugs and result in increased patient benefit.

This study exhibited some limitations. First, to study genes related to disease severity, we excluded patients whose SLEDAI scores remained stable over time. However, only four such patients were excluded, which would have had minimal impact on the results. Second, due to limitations of the data and since most patients in our study experienced combined treatment, the evaluation of drug treatment might have been affected to a certain extent. Also, when analyzing the therapeutic effect of IFN-K on each subtype, we could not accurately exclude the influence of dose and other confounding factors on the results due to data limitations. Subsequent assessments will further test our findings, improve our methods, and integrate multiple omics data to establish a more complete model mechanism that can be accurately applied in clinical trials.

In summary, we have defined three different clinical subtypes of SLE, which have different functional features. For the first time, we have fully revealed the functional mechanisms of SLE subtypes and determined their potential interaction-mediated roles. In addition, we have demonstrated that patients with distinct SLE subtypes benefit from different drugs, which will guide the choice of therapeutic drugs more rationally, accelerate the development of new drugs for particular subtypes, and reposition targeted drugs within subtypes.

## Data Availability

The data sets (GSE65391, GSE49454, and GSE72754) were used in this paper from GEO database.

## Supplementary Materials

Supplementary Materials Using R (version 4.0.2) statistical software to process and analyze numerical data. A detailed description of the score generation, SSC development, and modular analysis is provided in the supplementary material.
