# Supplementary material for: Blood Genomics Identifies Three Subtypes of Systemic Lupus Erythematosus: “IFN-High,” “NE-High,” and “Mixed”
Source: Mediators Inflamm. 2021 Jul 1;2021:6660164. doi: 10.1155/2021/6660164 (PMC8270691; doi:10.1155/2021/6660164)
Supplement: Supplementary Materials — Using R (version 4.0.2) statistical software to process and analyze numerical data. A detailed description of the score generation, SSC development, and modular analysis is provided in the supplementary material. [file 6660164.f1.docx]

**Supplementary Information**

**Determination of the molecular subtypes in SLE and study of its mechanism**

**SLE patients**

Two patient groups based on the same platform were downloaded from GEO: adult samples (GSE49454) and pediatric samples (GSE65391). In order to minimize the chip-to-chip variability, we screened the probes and preprocess the data by applying quantile normalization.

**Score generation**

(1) Training parameters to evaluate the contribution of each patient to the score. In order to evaluate the contribution of each patient’s data to the correlation between genes and SLE, we calculated the correlation between genes and SLEDAI in each patient, and calculated the correlation after the scramble. We performed a paired rank sum test for correlation, loop 1000 times, and used significantly different times as a parameter for each patient. (2) Calculating the correlation score between genes and SLE. In order to verify the overall score, we randomly shuffled the data and generated a series of pseudo samples. We calculated each pseudo sample to get a series of scores, compared score of each group with the score of original data, and got the overall distribution of p-value. The significant number of the P-value is greater than 95% proves that the score is specific and the parameters are reliable. (3) Verifying the overall score. We selected 113 non-difference genes, and calculated the overall score of 226 genes. We performed a rank sum test on differential genes and non-differential genes, counted the number of times the p value is significant, and 1000 times were all positive, which proved that our score is reliable.

**SSC development**

Three clustering algorithms are used to determine the optimal number of subtypes and types of the sample:

(1) The Calinski-Harabasz criterion is called the variance ratio criterion (VRC), which can be used to determine the optimal K value for clustering. Therefore, if the is smaller and the is larger, the clustering effect will be better, that is, the larger the Calinsky criterion value, the better the clustering effect.

(2) PAM (Partitioning Around Medoids): partitioning algorithm around the center point. Select the most central object in the cluster, and try to give k divisions to n objects; the representative object is also called the center point, and other objects are called non-representative objects; initially, k objects are randomly selected as the center point, The algorithm repeatedly replaces representative objects with non-representative objects, trying to find a better center point to improve the quality of clustering; in each iteration, all possible pairs of objects are analyzed, one object in each pair Is the central point, and the other is the non-representative object. For possible combinations, estimate the quality of the clustering results; an object can be replaced by an object that reduces the maximum square-error value; the best object set produced in one iteration becomes the center point of the next iteration.

(3) NBClust is a comprehensive method1. The NbClust software package provides 30 indicators for determining the number of clusters, and is obtained different results by changing all combinations of the number of clusters, distance metrics and clustering methods, and propose the best clustering scheme to the user.

We used consensus class to initially determine the subtype, calculated the subtype feature value, and further correct the subtype: (1) Calculating the feature value of each subtype. We calculated the central value of each gene and the shortest total distance for each sample; (2) Classify the subtypes with the feature values​of 92 genes of each subtype. We calculated the total absolute distance from each sample to the feature value, corrected the subtypes that have been classified, and recalculated each feature value of the subtypes; (3) Loop operation, it was found that after the sixth correction, the model converged when only a few samples were changed.

**Modular function analysis**


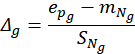
For a biological process, we calculated the expression of the genes contained in the biological process
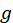
 in the subtype and the difference between the patient and the health gold standard, and then calculated the average value of the absolute value to obtain the expression value of
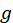
:

Where
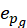
 is the expression level for patient p of biological process g,
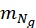
 is the mean expression level of biological process g for the normal samples, and
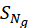
 is the standard deviation of the expression levels across the normal samples.

We summarized BPs into five major modules: virus infection (virus, MDA5, ISG15, RIG1 signaling pathway), bacterial infection (bacteria, gram-positive bacteria, gram-negative bacteria), fungal infection, IFN, immune disorder, and compared the imbalance of different module subtypes.

We also analyzed the modules in IFN-high and NE-high. In the IFN-high, it was found that virus is recognized by the receptor and triggers an antiviral interferon response. Excessive IFN then leads to the release of pro-inflammatory cytokines, leading to autoimmunity. In the NE-high, bacteria and fungus are found to be recognized by receptors and produce pro-inflammatory cytokines and AMP, which cause NE degranulation, leading to the production of NETs and SLE.
